# Ang-(1-7) is an endogenous β-arrestin-biased agonist of the AT_1_ receptor with protective action in cardiac hypertrophy

**DOI:** 10.1038/s41598-017-12074-3

**Published:** 2017-09-19

**Authors:** Larissa B. Teixeira, Lucas T. Parreiras-e-Silva, Thiago Bruder-Nascimento, Diego A. Duarte, Sarah C. Simões, Rafael M. Costa, Deisy Y. Rodríguez, Pedro A. B. Ferreira, Carlos A. A. Silva, Emiliana P. Abrao, Eduardo B. Oliveira, Michel Bouvier, Rita C. Tostes, Claudio M. Costa-Neto

**Affiliations:** 1 Department of Biochemistry and Immunology, Ribeirao Preto Medical School, University of São Paulo, Ribeirao Preto, SP 14049-900 Brazil; 2 Department of Pharmacology, Ribeirao Preto Medical School, University of São Paulo, Ribeirao Preto, SP 14049-900 Brazil; 3 Department of Biochemistry and Molecular Medicine and Institute for Research in Immunology and Cancer, University of Montréal, Montréal, QC H3C-3J7 Canada

## Abstract

The renin-angiotensin system (RAS) plays a key role in the control of vasoconstriction as well as sodium and fluid retention mediated mainly by angiotensin (Ang) II acting at the AT_1_ receptor (AT1R). Ang-(1-7) is another RAS peptide, identified as the endogenous ligand of the Mas receptor and known to counterbalance many of the deleterious effects of AngII. AT1R signaling triggered by β-arrestin-biased agonists has been associated to cardioprotection. Because position 8 in AngII is important for G protein activation, we hypothesized that Ang-(1-7) could be an endogenous β-arrestin-biased agonist of the AT1R. Here we show that Ang-(1-7) binds to the AT1R without activating Gq, but triggering β-arrestins 1 and 2 recruitment and activation. Using an *in vivo* model of cardiac hypertrophy, we show that Ang-(1-7) significantly attenuates heart hypertrophy by reducing both heart weight and ventricular wall thickness and the increased end-diastolic pressure. Whereas neither the single blockade of AT_1_ or Mas receptors with their respective antagonists prevented the cardioprotective action of Ang1-7, combination of the two antagonists partially impaired the effect of Ang-(1-7). Taken together, these data indicate that Ang-(1-7) mediates at least part of its cardioprotective effects by acting as an endogenous β-arrestin-biased agonist at the AT1R.

## Introduction

The renin-angiotensin system (RAS) is a critical regulator of cardiovascular and renal physiology, controlling among other functions, blood pressure, electrolyte balance and cardiac remodeling^1^. The RAS cascade starts with angiotensinogen, a large protein, mainly produced by the liver, that is cleaved by the enzyme renin, generating the decapeptide angiotensin I (AngI, sequence: Asp-Arg-Val-Tyr-Ile-His-Pro-Phe-His-Leu). AngI is an inactive intermediate that serves as a substrate for different enzymes generating distinct active or inactive peptides^2^. For instance, the angiotensin converting enzyme (ACE) processes AngI to generate the octapeptide AngII (sequence: Asp-Arg-Val-Tyr-Ile-His-Pro-Phe), which is then cleaved by ACE2 or other carboxypeptidases to generate the heptapeptide Ang-(1-7) (sequence: Asp-Arg-Val-Tyr-Ile-His-Pro). The direct action of endopeptidases, such as the thimet oligopeptidase, on AngI has also been reported to produce Ang-(1-7)^3–6^.

AngII binds to AngII type 1 (AT_1_) and type 2 (AT_2_) receptors, which belong to the G protein-coupled receptors (GPCRs) superfamily. Binding of AngII to the AT_2_ receptor has not been reported to activate any of the known G proteins or their canonical downstream effectors^7^. On the other hand, binding of AngII to the AT_1_ receptor (AT1R) triggers Gq activation leading to phospholipase C (PLC) stimulation, production of inositol trisphosphate (IP_3_) and diacylglycerol (DAG), intracellular calcium (Ca^2+^) mobilization, activation of protein kinase C (PKC) and downstream cellular effectors. In addition, binding of AngII to AT1R has also been reported to activate Gi/o and G12/13^8^. AT1R activation accounts for most of the classical actions of AngII, including vasoconstriction, sodium and water reabsorption as well as cell growth, proliferation, and matrix deposition^9^.

Ang-(1-7) was described as a pharmacologically active peptide^10^ before it was identified as an endogenous ligand of the Mas receptor^11^, an orphan receptor that also bears the characteristic GPCR seven transmembrane domain, and that until then was known as a proto-oncogene^12,13^. Activation of the Mas receptor by Ang-(1-7) has not been reported to stimulate any of the known G proteins, but has been shown to increase arachidonic acid levels and to trigger Akt-dependent pathways. Interestingly, from a pathophysiological perspective, activation of the Mas receptor by Ang-(1-7) has been reported to counterbalance AngII-induced negative cardiovascular effects^14^.

Concerning the AT1R, it is well known that the last C-terminal amino acid residue (Phe^8^) of AngII plays a pivotal role in the agonistic properties of the ligand^15,16^, mainly by affecting the activation of G protein signaling cascades. For instance, ligands such as SII and TRV027 that harbor modifications at the Phe^8^ position show reduced activation of G protein signaling while maintaining their ability to promote β-arrestin recruitment and signaling^17,18^. Based on these observations, we hypothesized that Ang-(1-7) could bind to the AT1R and act as an agonist with distinct functionalities, such as β-arrestin-biased agonist properties. To address that, we used a heterologous system expressing the AT1R, where we observed that indeed Ang-(1-7) binds to this receptor, does not lead to G protein activation, but robustly induces β-arrestins 1 and 2 recruitment and activation, and triggers ERK1/2 phosphorylation. This profile of activation reveals a rare signature of a “pure” endogenous β-arrestin-biased agonist. Further to that, we performed *in vivo* experiments which showed that Ang-(1-7) infusion attenuated left ventricular wall thickness and reduced end-diastolic pressure in a rat model of cardiac remodeling.

## Results and Discussion

### Ang-(1-7) binds to the AT_1_ receptor but does not engage its canonical G protein signaling

**Figure 1:**
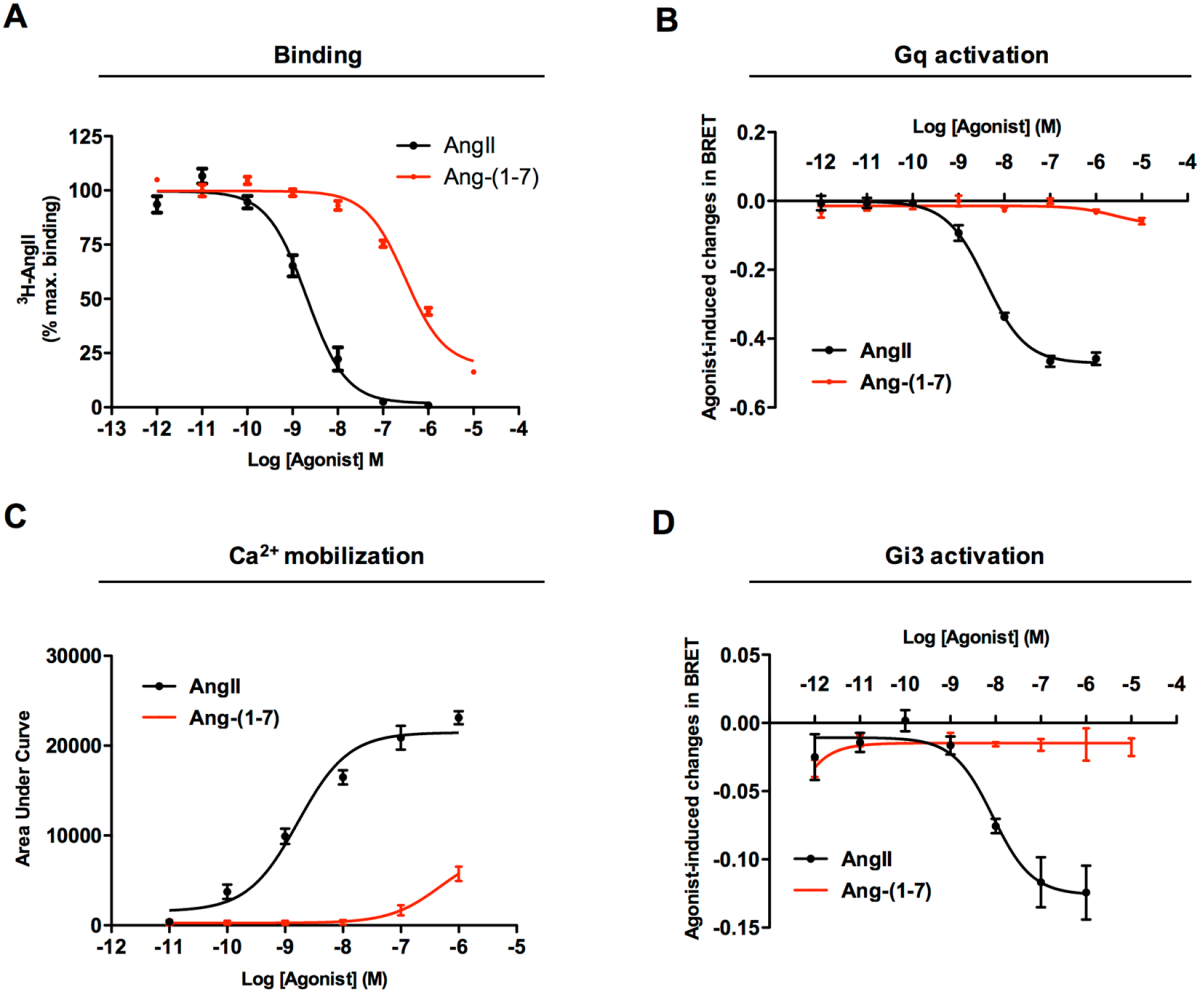
Comparative analyses of Ang-(1-7) and AngII acting at the AT_1_ receptor: binding and analyses of G protein activation pathways. (**A**) Competition binding profiles for AngII and Ang-(1-7) against [^3^H]AngII in HEK293T cells transiently expressing the AT1R. Data are expressed as percentages of the maximum specific binding of the radioligand. AngII and Ang-(1-7) effects on Gq activation (**B**), intracellular Ca^2+^ mobilization (**C**) and Gi3 activation (**D**). All data were generated from at least 3 independent experiments.

To test the ability of Ang-(1-7) to bind to the AT1R we performed competition binding assays using [^3^H]AngII as the radiolabeled ligand and Ang-(1-7) or AngII as competitors. Figure 1A shows that Ang-(1-7) indeed binds to the AT1R with a characteristic profile for competition binding assays, yielding an affinity of ~200 nM, while AngII’s affinity is ~2 nM, as expected (Table 1).

**Table 1:** Affinities of AngII and Ang-(1-7) for the AT_1_ receptor from competition binding assays, and functional data presented as potencies (pEC_50_) and efficacies (E_max_) in promoting G protein- dependent signaling pathways.

	Binding	G_q_ activation		Ca^2+^ mobilization		G_i3_ activation	
	pK_i_	pEC_50_	E_max_ (%)	pEC_50_	E_max_ (%)	pEC_50_	E_max_ (%)
AngII	8.81 ± 0.25	8.39 ± 0.10	100	8.75 ± 0.14	100	8.11 ± 0.25	100
Ang-(1-7)	6.66 ± 0.14	N.D.	12.8	N.D.	24.8	N.D.	N.D.

N.D.: Values could not be determined. EC_50_ values are presented as mean ± SEM of at least three independent experiments. E_max_ values for Ang-(1-7) are shown as a percentage of the maximum values obtained for AngII.

Although Ang-(1-7) lacks the Phe^8^ residue, predicted to be important for the binding of AngII to the AT1R^15,16^, its binding to the receptor has been previously described. However, different studies reported different affinities of Ang-(1-7) for the AT1R, varying from high^19^ to low^20,21^, or no interactions^22^. The lack of apparent binding observed in previous reports could be due to the radioligand used in some of these studies (i.e: [^125^I]Sar^1^Ile^8^-AngII vs. [^3^H]AngII), or to the presence of other GPCRs, since interactions with different receptors have been suggested to affect AT1R properties^23–28^.

To assess whether the binding of Ang-(1-7) to AT_1_ could promote activation and downstream signaling, we initially monitored heterotrimeric G protein activation using bioluminescence resonance energy transfer (BRET)-based biosensors, which monitor proximity between an energy donor and an acceptor attached to proteins of interest^29^. Following G protein activation, the Gα subunit separates from the Gβγ subunits, which in our approach is detected as a decrease in BRET signal between Gα subunit fused to Renilla luciferase (Rluc) and Gγ fused to GFP_10_^30^. The classical signaling pathway triggered by AngII stimulation of the AT1R involves the Gq subtype of G proteins. As shown in Fig. 1B, Ang-(1-7) did not promote the separation of Gαq-RLucII from Gγ1-GFP_10_ at any of the concentrations tested in HEK293T cells transiently expressing AT1R. This contrasted with the activation of Gq promoted by AngII, reflected by the dose-dependent decrease in BRET signal with a potency of 4.25 nM (Fig. 1B, Table 1).

The signaling cascade downstream of Gq starts with the activation of PLC, which hydrolyses phosphatidylinositol (4,5)-bisphosphate (PIP_2_) to generate DAG and IP_3_, with the later leading to increase of intracellular Ca^2+^ ^31–33^. We thus then examined intracellular Ca^2+^ mobilization induced by both ligands in HEK293T cells transiently expressing AT1R. As illustrated by the concentration-response curve (Fig. 1C), only a weak increase in intracellular Ca^2+^ levels was observed after stimulation with Ang-(1-7). Given that Ca^2+^ mobilization represents an amplified signal, the weak signal indicate that only a very poor, if any, activation of G-protein mediated calcium mobilization can be promoted by Ang-(1-7). As expected, AngII promoted a robust concentration dependent Ca^2+^ mobilization with a potency of ~2 nM (Fig. 1C, Table 1).

Since AngII has been previously reported to activate Gi^34^, we also evaluated Gi3 protein activation using a BRET-based biosensor. As shown in Fig. 1D, Ang-(1-7) did not trigger Gi3 activation, whereas AngII produced a concentration-dependent activation of Gi3 with a potency of ~10 nM (Fig. 1D, Table 1) confirming that, in addition to activate Gq, AT1R can activate Gi protein family members when activated by AngII but not upon stimulation with Ang1-7.

### Ang-(1-7) promotes AT1R engagement and activation of β-arrestins 1 and 2 and triggers ERK1/2 phosphorylation

**Figure 2:**
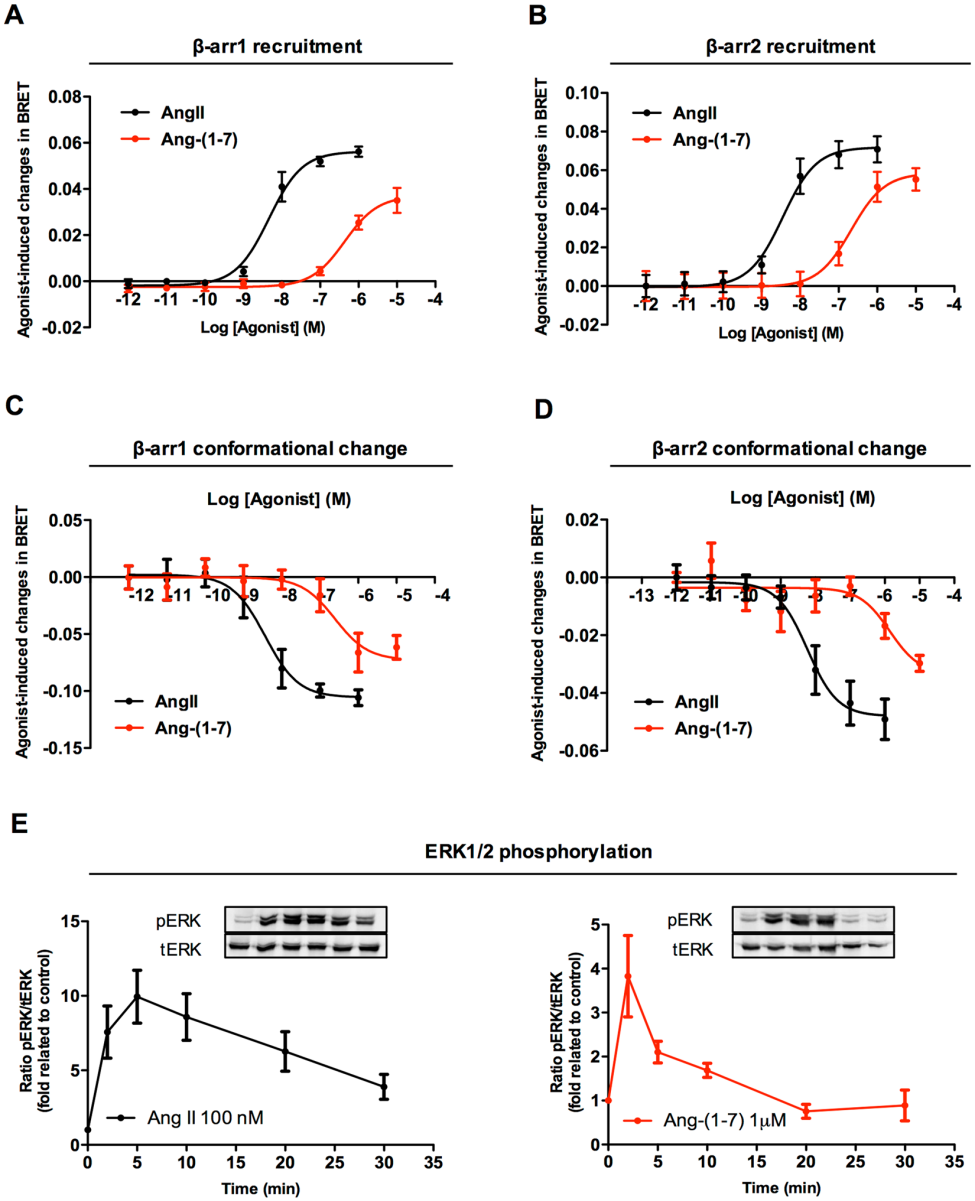
Comparative analyses of Ang-(1-7) and AngII acting at the AT_1_ receptor to induce β-arrestins recruitment, β-arrestins conformational changes, and ERK1/2 phosphorylation. Concentration-response curves for Ang-(1-7) and AngII were generated to evaluate the recruitment of β-arrestin 1 (**A**) or β-arrestin 2 (**B**) to the AT1R by BRET. The abilities of the ligands to trigger β-arrestin activation, as evaluated by conformational rearrangements of β-arrestin 1 (**C**) or β-arrestin 2 (**D**), were monitored by intramolecular BRET as described in Methods. Kinetics of ERK1/2 phosphorylation after stimulation with 100 nM AngII or 1 μM Ang-(1-7) (**E**). Data were generated from at least 4 independent experiments. Blots were cropped for conciseness of the presentation; full-length blots are presented in Supplementary Figure S1.

To assess whether Ang-(1-7) promotes β-arrestin engagement and activation, we first monitored the recruitment of β-arrestin-1 and -2 to AT1R in response to either Ang-(1-7) or AngII by monitoring the agonist promoted BRET between β-arr1-RLucII or β-arr2-RLucII and AT_1_R-GFP (Fig. 2A,B). A robust concentration dependent increase in BRET signal was detected for both β-arrestins after receptor stimulation with either Ang-(1-7) or AngII. The potency for each of the ligand was compatible with the respective affinity of the peptides for AT1R, Ang-(1-7) having a significantly lower potency than AngII. However, Ang1-7 was quite efficacious in promoting β-arrestin recruitment, reaching almost 70% and 90% of the AngII maximal responses for β-arrestin-1 and -2, respectively (Fig. 2A and B, Table 2). Therefore, although Ang-(1-7) cannot promote sizable AT1R activation of Gq or Gi3, it is an efficient partial agonist for β-arrestin engagement, indicating that it is a β-arrestin biased agonist at the AT1R.

**Table 2:** Potencies (pEC_50_) and efficacies (E_max_) of AngII and Ang-(1-7) in promoting β-arrestin 1 and β-arrestin 2 recruitment and conformational changes.

	β-arr1 recruitment		β-arr2 recruitment		β-arr1 conf. changes		β-arr2 conf. changes	
	pEC_50_	E_max_ (%)	pEC_50_	E_max_ (%)	pEC_50_	E_max_ (%)	pEC_50_	E_max_ (%)
AngII	8.29 ± 0.15	100	8.42 ± 0.08	100	8.43 ± 0.23	100	8.37 ± 0.14	100
Ang-(1-7)	6.38 ± 0.14	67.8	6.56 ± 0.09	87.7	6.61 ± 0.38	69.1	5.86 ± 0.53	64.4

EC_50_ values are presented as mean ± SEM of at least three independent experiments. E_max_ values for Ang-(1-7) are shown as a percentage of the maximum values obtained for AngII.

Receptor promoted activation of β-arrestin results in a conformational rearrangement that can be monitored by BRET-based double brilliance biosensors assessing changes in BRET between RLucII and GFP_10_ fused respectively to the N- and C-termini of β-arrestins^35–37^. Therefore, to assess whether the recruitment of β-arrestins to the AT1R by Ang-(1-7) leads to activation, we monitored the agonist-promoted change in the intramolecular BRET. As illustrated in Fig. 2C and D, both AngII and Ang-(1-7) promoted concentration-dependent decrease in BRET that reflected β-arrestin-1 and -2 activation. As was the case for the agonist-promoted recruitment, both peptides promoted β-arrestin conformational rearrangement with potencies corresponding to their affinities, with Ang-(1-7) behaving as a strong partial agonist as compared to AngII (Table 2).

Given the known role of β-arrestin in AT1R-promoted ERK1/2 phosphorylation, we then assessed whether Ang-(1-7) could promote ERK1/2 activation via AT1R. As can be seen in Fig. 2E, both AngII and Ang-(1-7) promoted ERK1/2 phosphorylation; however, as was the case for the recruitment and activation of β-arrestins, Ang-(1-7) behaved as a partial agonist resulting in lower and more transient activation. We believe that phosphorylation of ERK1/2 in HEK293 cells expressing the AT1R by Ang-(1-7) is a pivotal finding, as it can be linked to previous reports that correlate β-arrestin-biased activation and ERK1/2 phosphorylation to cardioprotective effects^38,39^.

Altogether, these data clearly show that Ang-(1-7), classically known as the Mas receptor endogenous agonist^11^, also acts as a β-arrestin-biased agonist on the AT1R resulting in a signaling profile distinct from AngII. Interestingly, the observed β-arrestin-biased profile resembles that reported for molecules with cardioprotective effects in heart failure animal models^40,41^, such as the synthetic TRV027^18^. TRV027 is a β-arrestin-biased agonist of the AT1R that until recently was in Phase 2b clinical trials for treatment of acute heart failure (AHF)^42^. Last year, Trevena announced that TRV027 failed to meet the primary or secondary efficacy endpoints of the clinical trial (Trevena, Inc., press release; http://www.trevena.com/news-details.php?id=145). Although the detailed reasons for the failure are still unclear, no significant safety concerns were raised by the study, and the potential cardioprotective action of β-arrestin activation has been recently reinforced^43^. While this manuscript was in preparation, a publication from an independent group also reported that Ang-(1-7) acts as an arrestin biased agonist at the AT1R, consistent with the data presented herein^44^. As Ang-(1-7) does not stimulate vasoconstriction, or sodium and fluid retention, and may even block these AngII-promoted cardiodeleterious events, while increasing cardiomyocyte contractility and protecting against cardiac cell apoptosis (effects that have been linked to beneficial AT1R promoted β-arrestin engagement^43^), we investigated its possible *in vivo* cardiac actions through the AT1R^18,45–48^. Although one could argue about the low affinity of Ang-(1-7) to AT1R as compared to AngII, we believe that Ang-(1-7) is likely to have a more preponderant effect in tissues/organs where it is locally or paracrinally produced, allowing therefore higher concentrations to be available^49^. Interestingly, we have recently reported that Ang-(1-7) is the main AngI-derived peptide formed in the hippocampus, where AngII is in fact not formed^5^.

To investigate whether AT1R signaling contributes to the cardioprotective response associated with Ang-(1-7)^34^, we used an experimental model of cardiac hypertrophy that does not rely on AngII-mediated mechanisms^50^. To our knowledge, this is the first *in vivo* study that addressed possible Ang-(1-7) cardioprotective effects when acting as a β-arrestin-biased agonist of the AT1R.

### Ang-(1-7)-induced reduction of cardiac hypertrophy and of end-diastolic pressure involves both AT_1_ and Mas receptors

**Figure 3:**
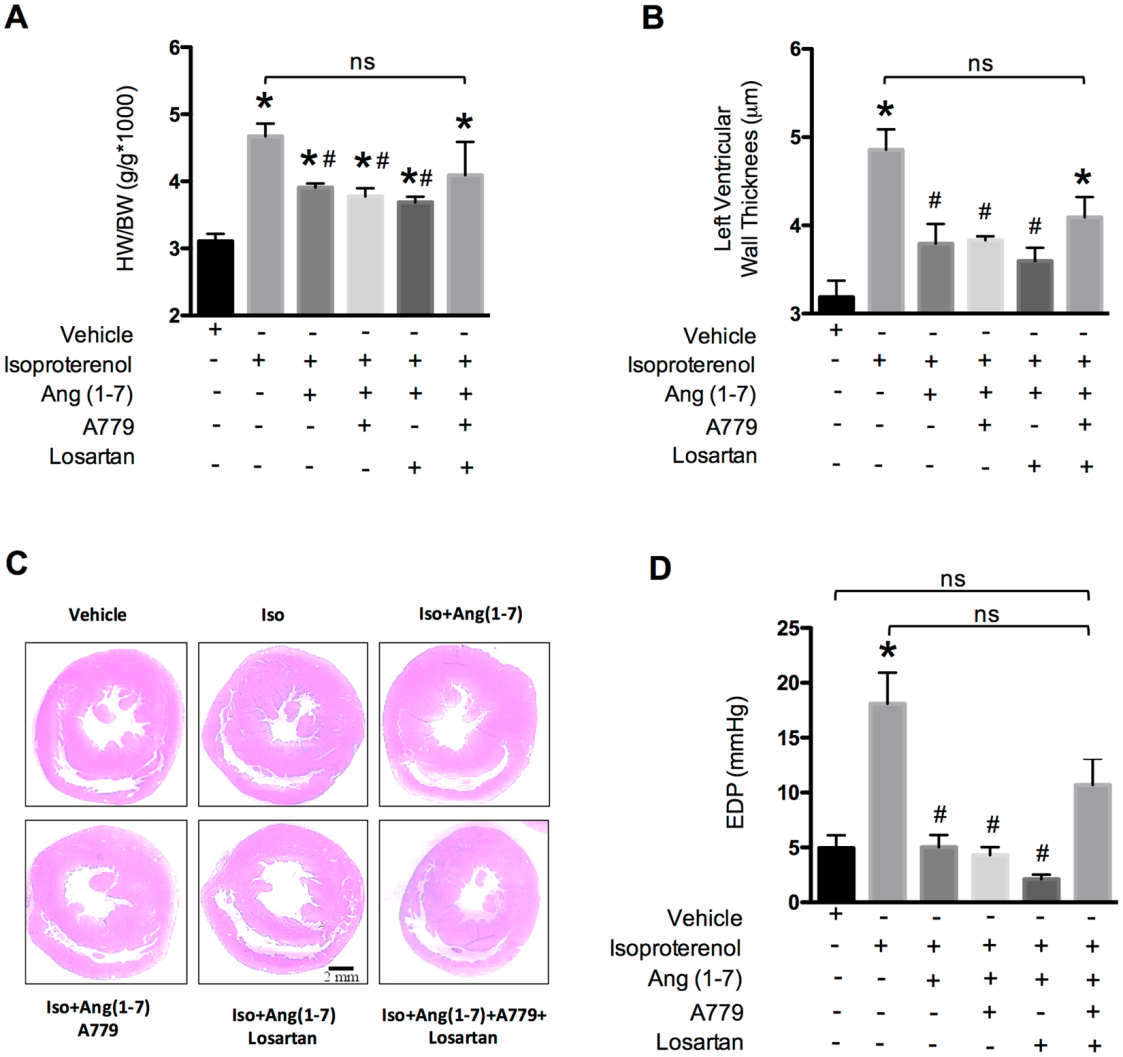
Effect of Ang-(1-7) on cardiac hypertrophy and increased end-diastolic pressure induced by isoproterenol. Cardiac hypertrophy was determined by heart weight/body weight ratio (HW/BW) (**A**), left ventricular wall thickness (**B,C**), and end-diastolic pressure (EDP) (**D**). Representative images of hearts from different groups of treatment stained with hematoxylin and eosin are shown in **C**. Wistar rats were treated with either vehicle or Isoproterenol (Iso) (2 mg/kg per day, i.p. for 7 days). Rats were also treated for 7 days with Ang-(1-7) (24 μg/kg per hour, Alzet osmotic mini-pump), Ang-(1-7) + losartan (AT1R selective antagonist, 10 mg/kg per day orally), Ang-(1-7) + A779 (Mas receptor selective antagonist, 744 μg/kg per day i.p.), or Ang-(1-7) + Losartan + A779 (same doses as described). Scale bars in the representative heart images = 2 mm. Bars represent the mean ± SEM (n = 6 vehicle, n = 6 Iso, n = 4 Iso + Losartan, n = 4 Iso + A779, n = 4 Iso + A779 + Losartan). *P < 0.05 vs. control; ^#^P < 0.05 vs. Iso treatment. ns: not significant.

**Figure 4:**
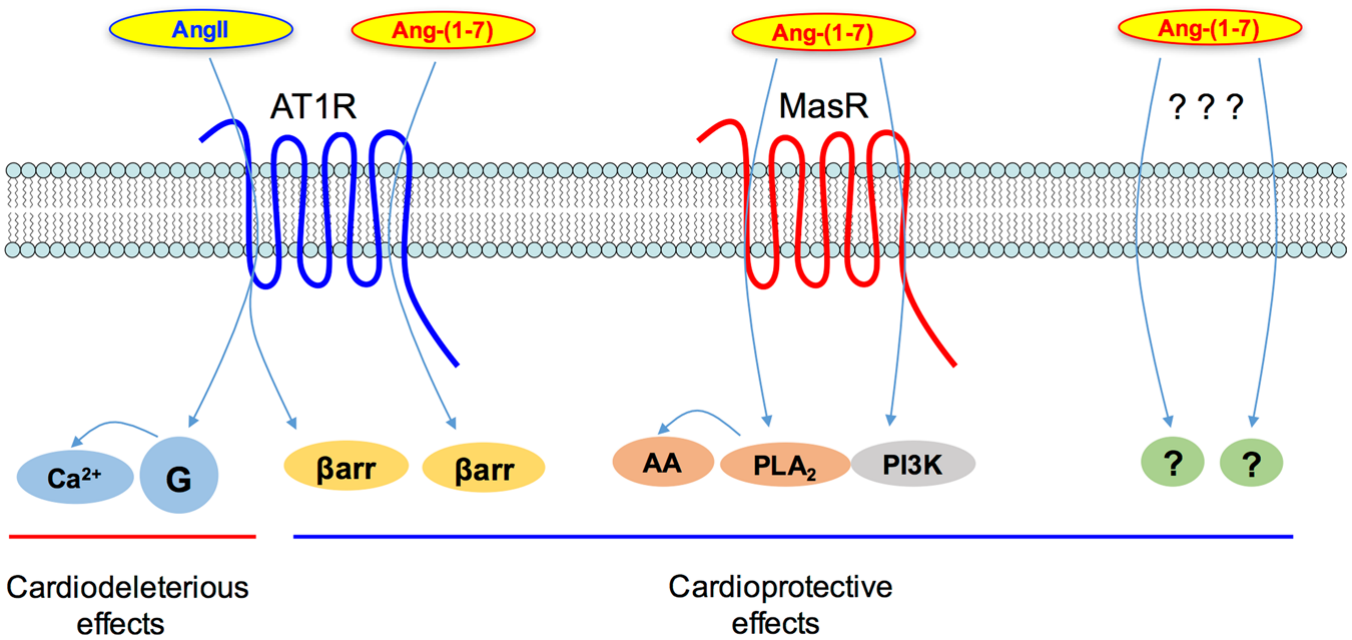
Schematic representation of the possible mechanism by which Ang-(1-7) plays its protective role in cardiac hypertrophy. AT1R is the main receptor for AngII, a GPCR coupled to G_q_ that leads to calcium release and modulation of signaling pathways related to cardiodeleterious effects, including cardiac hypertrophy. Besides G protein activation, AngII binding to AT1R also leads to β-arrestin recruitment, impairing G protein signaling and inducing receptor internalization. AT1R coupling to β-arrestin also initiates a subset of arrestin-dependent signaling pathways, which have been involved in cardioprotective effects. Ang-(1-7) is known as the Mas receptor endogenous ligand, reported to trigger some of its signal transduction by activation of PLA2/AA and PI3K/AKT^11,68–70^, exerting physiological outcomes that counteract the AngII/AT1R cardiodeleterious effects. Here we show that Ang-(1-7) also binds to AT1R, but in a β-arrestin biased fashion, leading to attenuation of cardiac hypertrophy. These data suggest that some of the known cardioprotective Ang-(1-7) effects may also occur due to activation of AT1R in a β-arrestin-biased way. Indeed, our data show that the individual blockage of either AT1R or Mas receptor with their selective antagonists was not able to revert the cardioprotective effects induced by Ang-(1-7). The combined use of both AT1R and Mas receptor antagonists partially reverted the Ang-(1-7) effects, suggesting the involvement of both receptors, but was still not able to fully revert them, indicating that Ang-(1-7) effects could depend upon its interaction with other targets.

In agreement with previous studies, isoproterenol treatment promoted cardiac hypertrophy, characterized by increased heart weight/body weight (HW/BW) ratio (Fig. 3A) and by increased left ventricular wall thickness, as evaluated by histomorphometric analysis (Fig. 3B and C), as well as increased end-diastolic pressure (EDP) (Fig. 3D). Treatment of animals with Ang-(1-7) was performed concomitantly with isoproterenol (see Methods), and partially blocked the development of cardiac hypertrophy (Fig. 3B) and HW/BW ratio (Fig. 3A), and completely prevented the increase in EDP (Fig. 3D). Very interestingly, co-treatment of the animals with Ang-(1-7) and only one of the antagonists, Losartan (selective antagonist of the AT1R) or A779 (selective antagonist of the Mas receptor^51^), did not prevent the effect of Ang-(1-7) on the cardiovascular parameters (Fig. 3A,B and D). However, the combination of both antagonists resulted in a partial blockade of the beneficial effects of Ang-(1-7), yielding values that are no longer significantly different from the isoproterenol-treated group (Fig. 3A,B and D). These data indicate that binding of Ang-(1-7) to either of the two receptors can elicit the beneficial action of Ang-(1-7) and that blockade of both receptors is required to at least partially block this action. Whether this observation may be linked to the proposal that AT1R and the Mas receptor can form heterodimers^26^ remains to be investigated. Indeed, the fact that the combination of the two antagonists at concentrations that would be predicted to fully block the two receptors did not completely block the physiological action of Ang-(1-7) suggests that AT1R/Mas receptor heterodimerization could generate an entity with altered pharmacological profile, or that additional receptor(s) are involved in Ang-(1-7) action (see Fig. 4). Further studies will be needed to address if such additional target may exist.

The involvement of Mas receptor in cardioprotection has been extensively reported in the literature^49,52–59^, where for instance its activation has been shown to attenuate heart failure induced by myocardial infarction^57^, to rescue cardiac function in diabetic rats^58^, and to decrease arterial pressure of spontaneously hypertensive rats^59^. Therefore, in addition to such known cardioprotective actions of the Mas receptor, our study unveils that Ang-(1-7) β-arrestin-biased mode of action at the AT1R may also play a key role on such protective actions.

## Methods

### Cell Culture and Transfection

All reagents and cell culture supplies, except where specified, were obtained from Invitrogen (Carlsbad, CA, USA); AngII was purchased from Sigma-Aldrich (St. Louis, MO, USA). HEK293T cells were cultured in DMEM supplemented with 10% heat-inactivated fetal bovine serum and 100 U/mL penicillin/streptomycin at 37 °C under 5% CO_2_. Cells were seeded in 100-mm dishes and 48 hours before the experiments, transiently transfected with the AT1R (alone or in combination with BRET-based biosensors). Transient transfections were performed using 25 kDa linear polyethylenimine (PEI – Polysciences, Warrington, PA, USA) at a ratio of 3:1 PEI/DNA. The total amount of transfected DNA was kept constant (10 μg/dish) by the addition of salmon sperm DNA.

### Binding assay in whole cells

Binding assays were performed in whole HEK293T cells at 48 hours after transfection. Twenty-four hours before the experiment, transfected cells were transferred to 24-well plates, treated with poly-l-lysine, and then incubated overnight at 37 °C, in a 5% CO_2_ environment. Before the binding assays, cells were washed with wash buffer (25 mM Tris-HCl, pH 7.4, 140 mM NaCl, 5 mM MgCl_2_, 0.1% BSA) and incubated with cold binding buffer (25 mM Tris-HCl, pH 7.4, 5 mM MgCl_2_, 0.1% BSA (w/v), and 100 μg/mL bacitracin (Sigma-Aldrich, St. Louis, MO, USA). Tritiated AngII ([^3^H]AngII – American Radiolabeled Chemicals, St. Louis, MO, USA) at 0.5 nM in presence of different concentrations of competitor ligands, diluted in binding buffer, were added to a final volume of 525 μL and plates were incubated at 4 °C. Twenty-four hours later, wells were washed twice with wash buffer and incubated with 500 μL of lysis buffer (48% urea, 2% NONIDET P-40, prepared in 3 M acetic acid) for 15 minutes. Samples were collected and mixed with 3 mL of scintillation liquid, and radioactivity was measured using a Liquid Scintillation Counter (Packard Tri-Carb 2100TR – PerkinElmer).

### BRET Assays for G protein activation, β-arrestin recruitment and β-arrestin conformational change

HEK293T cells transiently expressing the AT1R (or AT_1_R-GFP) and BRET-based biosensors (Gαq-RLucII or Gαi3-RLucII, Gβ1 and Gγ1-GFP_10_; RLucII-β-arr1-GFP_10_; RLucII-β-arr2-GFP_10_; β-arr1-RLucII; β-arr2-RLucII) were washed once with PBS, detached, and seeded in 96-well white plates (OptiPlate – PerkinElmer, Waltham, MA, USA). Following agonist stimulation, cells were incubated at 37 °C and the luciferase substrate (coelenterazine H for BRET1 or coelenterazine 400a for BRET2 – Biotium, Hayward, CA, USA) was added 5 min before reading BRET in a Victor^TM^ X Light Luminescence microplate reader (PerkinElmer) equipped with different donor/acceptor emission filter sets. The BRET signal was determined as the ratio of light emitted by fluorescently-labeled biosensors, detected with energy acceptor filters (530 ± 20 nm for BRET1 or 515 ± 20 nm for BRET2), and light emitted by RLucII-tagged biosensors, detected with energy donor filters (480 ± 20 nm for BRET^1^ or 410 ± 40 nm for BRET2). The specific BRET signal was defined as the difference between the total BRET signals and the one obtained with RLucII alone. Three independent experiments with full concentration-response curves were performed in cells stimulated with AngII or Ang-(1-7).

### Intracellular Calcium Mobilization Assay

Twenty-four hours after transfection, HEK293T cells were washed once with PBS, detached, and seeded in 96-well clear-bottom black plates (Cellstar – Greiner Bio-One, Monroe, NC, USA) precoated with poly-l-lysine (Sigma-Aldrich, St. Louis, MO, USA). Cells were cultured in supplemented DMEM without phenol red (Gibco) at a density of 50,000 cells/well for an additional 24-hour period. Cells transiently expressing the AT1R were loaded with a Ca^2+^-sensitive dye (FLIPR® Calcium 5 Assay Kit – Molecular Devices, Sunnyvale, CA, USA) containing 2.5 mM probenecid and incubated for 1 hour at 37 °C and 5% CO_2_. The plates were then transferred to a FlexStation 3 microplate reader (Molecular Devices), and fluorescence was measured at excitation and emission wavelengths of 485 nm and 525 nm, respectively. To provide a baseline level, fluorescence was recorded every 2 seconds during 16 seconds. Following agonist stimulation, fluorescence continued to be recorded every 2 seconds for 90 seconds. Three independent experiments with full concentration-response curves were performed in cells stimulated with AngII and Ang-(1-7).

### ERK1/2 phosphorylation evaluation by Western blotting

Cells transiently expressing the AT1R were seeded on 6-well plates in fully supplemented DMEM and kept at 37 °C. After 24 hours, cells were serum starved for 16 hours and then stimulated with 100 nM Ang II or 1 μM Ang-(1-7) for 2, 5, 10, 20 and 30 minutes. Cells were lysed with lysis buffer consisting of 10 mM Tris-HCl, pH 7.5, 150 mM NaCl, 1 mM EDTA, 1mM EGTA, 0.1% SDS, 1% Nonidet P-40 and the following protease inhibitors: 2 mM PMSF, 100 µg/mL SBTI, 10 µg/mL leupeptin, 100 µg/mL aprotinin, 10 mM benzamidin and 2 mM sodium orthovanadate. Following homogenization at 4 °C, during 30 minutes, cellular lysates were centrifuged for 15 minutes (18,000 × g) at 4 °C and total protein from the supernatants was quantified by the Bradford method (Bio-Rad, Hercules, CA). After that, 50 µg of total protein were resolved in SDS-PAGE, transferred to nitrocellulose membranes and Western blotting was performed against total ERK (tERK) and phosphorylated ERK (pERK) (both antibodies from Santa Cruz Biotechnology, Santa Cruz, CA, USA; and molecular size markers from ThermoFisher Scientific, Catalog Number 26610). Blots were captured with ImageQuant 350 (GE Healthcare, Piscataway, NJ, USA). Densitometric values obtained after analyses with ImageJ program (http://rsb.info.nhi.gov/ij/) were used to calculate a ratio pERK/tERK, and corresponding results were plotted using GraphPad software (GraphPad, San Diego, CA) as fold of increase over the basal value obtained with vehicle-treated control.

### Isoproterenol-induced cardiac hypertrophy and treatment with Ang-(1-7) and AT_1_ and Mas receptors antagonists

The isoproterenol-induced cardiac hypertrophy experimental model was chosen aiming to avoid any involvement of the RAS, as it is known that other models such as pressure-overload, SHR, or mRen2 involve RAS components^50,60,61^. Cardiac hypertrophy was induced in male, 6 weeks-old Wistar rats by administration of Isoproterenol [(Iso) 2 mg/kg per day, i.p. diluted in saline, for 7 days]. All experimental protocols were performed in accordance with the Brazilian Council for Control of Animal Experimentation - “Conselho Nacional de Controle de Experimentação Animal” (CONCEA), Brazil; and approved by the Animal Ethical Committee from the Ribeirão Preto Medical School, University of São Paulo (CEUA).

Rats receiving Iso were randomly submitted to treatment for 7 days with either vehicle, Ang-(1-7) [24 μg/kg per hour, via ALZET® osmotic minipumps (Durect, Cupertino, CA, USA)], Ang-(1-7) plus Losartan (10 mg/kg per day orally), Ang-(1-7) plus A779 (744 μg/kg per day i.p.), or Ang-(1-7) plus Losartan plus A779 (same doses as described). Doses and administration methods of drugs were based in previously published literature^62–66^.

Cardiac hypertrophy was confirmed by cardiac mass and histomorphometric analyses. To determine cardiac mass, hearts were removed, dried and weighted. Heart weight (HW) was normalized by the body weight (BW) (HW/BW*1000). For histomorphometric analysis, hearts were cut transversely, fixed in paraformaldehyde (4%) for 24 hours, and kept in ethanol (70%) until dehydration, which consisted in sequential washes with 70, 80, 90 and 100% ethanol. Samples were cleared in xylene and embedded in paraffin. Transversal sections (6 µm) were obtained from the cardiac samples at intervals of 40 µm and stained with hematoxylin and eosin. Images were captured using an Olympus BK 50 microscope (Olympus Corporation of the Americas, Center Valley, PA, USA) equipped with a SPOT RT3 digital camera (Diagnostic Instruments, Inc., Sterling Heights, MI, USA) and measured with ImageJ software (NIH, USA).

### Evaluation of cardiac function by end-diastolic pressure (EDP)

Rats were anesthetized with isoflurane (5% for induction and 2–2.5% to maintain anesthesia). A microtip pressure-volume catheter (SPR-839, Millar Instruments, Houston, TX, USA) was inserted into the right carotid artery for measurement of the arterial pressure and then moved into the left ventricle, as previously described^67^. After stabilization, signals were continuously recorded using a pressure-volume conductance system (MPVS, Millar Instruments) connected to a Power Lab/4SP (ADI Instruments, Sydney, NSW, Australia) feeding the digital signal to an IBM end-diastolic pressure (EDP).

### Statistical Analysis

All data are expressed as mean ± SEM. One-way analysis of variance (ANOVA), followed by the Bonferroni’s post-test. Analyses were performed using Prism 6.0 software (GraphPad). A difference was considered statistically significant when P ≤ 0.05.

## Electronic supplementary material

Supplementary information
