# Supplementary material for: Ang-(1-7) is an endogenous β-arrestin-biased agonist of the AT1 receptor with protective action in cardiac hypertrophy
Source: Sci Rep. 2017 Sep 19;7:11903. doi: 10.1038/s41598-017-12074-3 (PMC5605686; doi:10.1038/s41598-017-12074-3)
Supplement: Supplementary file 1 — Supplementary information [file 41598_2017_12074_MOESM1_ESM.pdf]

Supplementary Information.

Ang-(1-7) is an endogenous β-arrestin-biased agonist of the AT_1 receptor with protective action in cardiac hypertrophy

Larissa B. Teixeira, Lucas T. Parreiras-e-Silva, Thiago Bruder-Nascimento, Diego A. Duarte, Sarah C. Simões, Rafael M. Costa, Deisy Y. Rodríguez, Pedro A. B. Ferreira, Carlos A. A. Silva, Emiliana P. Abrao, Eduardo B. Oliveira, Michel Bouvier, Rita C. Tostes, Claudio M. Costa-Neto.

Supplementary Figure S1.

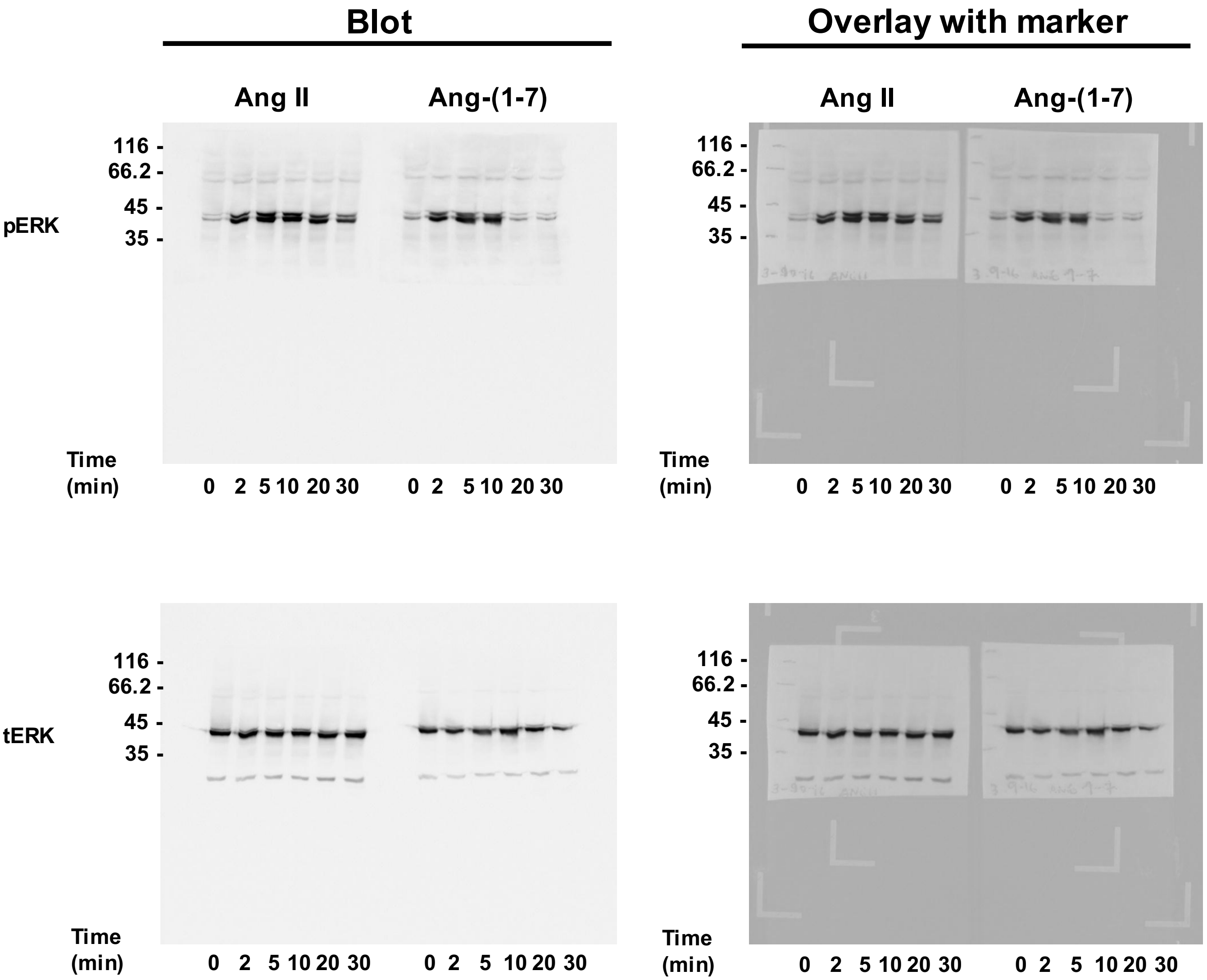

Figure S1. ERK1/2 phosphorylation evaluation by Western blotting. Western blotting was performed against total ERK (tERK) and phosphorylated ERK (pERK). Both antibodies were from Santa Cruz Biotechnology, Santa Cruz, CA, USA), and molecular size marker from ThermoFisher Scientific (Catalog Number 26610). Images were captured with ImageQuant 350 (GE Healthcare, Piscataway, NJ, USA).
